# RUASN: A Robust User Authentication Framework for Wireless Sensor Networks

**DOI:** 10.3390/s110505020

**Published:** 2011-05-04

**Authors:** Pardeep Kumar, Amlan Jyoti Choudhury, Mangal Sain, Sang-Gon Lee, Hoon-Jae Lee

**Affiliations:** 1 Department of Ubiquitous-IT, Graduate School of Design & IT, Dongseo University, Sasang-Gu, Busan 617-716, Korea; E-Mails: pradeepkhl@gmail.com (P.K.); choudhuryamlanjyoti@gmail.com (A.J.C.); mangalsain1@gmail.com (M.S.); 2 Division of Computer & Information Eng, Dongseo University. San 69-1, Jurye-2-Dong, Sasang-Gu, Busan 617-716, Korea; E-Mail: hjlee@dongseo.ac.kr

**Keywords:** wireless sensor network security, user authentication, user anonymity, session key establishment, confidentiality

## Abstract

In recent years, wireless sensor networks (WSNs) have been considered as a potential solution for real-time monitoring applications and these WSNs have potential practical impact on next generation technology too. However, WSNs could become a threat if suitable security is not considered before the deployment and if there are any loopholes in their security, which might open the door for an attacker and hence, endanger the application. User authentication is one of the most important security services to protect WSN data access from unauthorized users; it should provide both mutual authentication and session key establishment services. This paper proposes a robust user authentication framework for wireless sensor networks, based on a two-factor (*password and smart card*) concept. This scheme facilitates many services to the users such as user anonymity, mutual authentication, secure session key establishment and it allows users to choose/update their password regularly, whenever needed. Furthermore, we have provided the formal verification using Rubin logic and compare RUASN with many existing schemes. As a result, we found that the proposed scheme possesses many advantages against popular attacks, and achieves better efficiency at low computation cost.

## 1. Introduction

Wireless sensor networks (WSNs) are becoming more and more popular in everyday life as they offer economically viable, real time monitoring solutions. These wireless sensors can be quickly and easily deployed in hostile environments, and WSNs are now widely used in a variety of real-time applications, such as vehicular tracking, habitat monitoring, environment control, military surveillance, healthcare monitoring, wildlife monitoring and traffic monitoring. One recent survey declared that, in the near future, WSNs will become an intelligent and integral part of daily lives [1].

A WSN consists of a discrete group of independent, low cost, low power nodes with limited memory and computation power. They communicate wirelessly over limited frequency and low bandwidth [1]. More specifically, sensor nodes collectively monitor the area and sense substantial amounts of data, which are transmitted to the base-station traversing some nodes via RF signals and routing schemes.

As sensor nodes are resource constrained devices and are often deployed in a hostile environment and have to sense the information properly and efficiently. So the potential deployment of WSNs for any real-time applications has to deal with many challenges, including security, system architecture and protocol functionalities. Providing security to these resource hungry sensor networks is a very tedious task as compared to conventional networks, such as local area networks (*LANs*) and wide area networks (*WANs*). Consequently, providing suitable security has emerged as one of the critical issue in wireless sensor networks, and the state-of-art should therefore pay attention to how to deploy user-friendly, reliable and secure WSNs.

In real-time WSNs, sensor data queries are commonly issued from the base-station nodes or the backend application system. Moreover, these sensor networks can be accessed from anywhere in an *ad-hoc* manner. As sensor nodes provide services to users by themselves, it is necessary to control who is accessing the information and whether the intended user is authenticated to do so. Therefore, access control is a core requirement for WSNs to protect the data from access by unauthorized parties. In general user authentication where each user must verify their legitimacy is considered as one of the basic solutions for the access control issue.

So far, a number of significant schemes that provide adequate security for wireless sensor networks at the link layer [2–6] and the network layer [7] have been proposed. However, secure user authentication at the application layer has not been addressed effectively in order to prevent illegal access to sensor data. A review of current literature on WSNs reveals that only a few user authentication schemes have been adequately addressed [8–22] at the application layer. In [8–14], protocols are based on traditional passwords, where secrets are stored at the base station or the gateway. While, the protocols presented in [18–21] are based on two-factor user authentication with limited functionality (e.g., *no mutual authentication*, *no secure session key*, and *no confidentiality*) at high computation cost. Therefore, one of the primary concerns in wireless sensor network applications is the design and development of a robust user authentication scheme which is suitable for hostile or unattended environments.

In this paper, we have considered the above challenges and present a robust user authentication framework for wireless sensor networks (RUASN) at the application layer, which uses the two factor approach. The first factor (*something you know*) refers to something that is known by the user, such as a password, while the second factor (*something you have*) refers that something that is embedded on a device, such as smart cards, software tokens, digital certificates or biometric identifiers (e.g., *fingerprint scans* and so on) [23].

The proposed RUASN framework achieves user authentication (*access control*) for wireless sensor networks, where a user must login with same identity. The proposed scheme is resists many popular attacks, such as replay attack, impersonation attack, insider attack, stolen-verifier attack, password guessing attack, and man-in-the-middle attack. RUASN provides user privacy protection (*i.e.*, *user anonymity*), mutual authentication, and secure session key establishment. In addition, a user can update his/her password whenever demanded. Our framework uses one-way hash functions along with XOR operations to attain low computational overheads. Moreover, this paper further demonstrated the analysis and verification of the proposed protocol using Rubin Logic [24], which is very close to actual implementation.

The rest of the paper is structured as follows: Section 2 briefly reviews the related literature and the perceived weaknesses of existing schemes. In Section 3 we discuss the design goals, security requirements and system architecture of RUASN. In Section 4, we propose a robust two-factor user authentication framework in detail. Section 5 discusses the nonmonotonic cryptographic protocol and formal verification of proposed protocol using Rubin Logic. Section 6 discusses the security analysis, efficiency evaluation and comparison with existing schemes for wireless sensor networks. Finally, Section 7 conclusions are drawn for RUASN.

## 2. Literature Review

In this section, we will discuss the literature on user authentication schemes that have been recently proposed to verify the legitimacy of wireless sensor networks users.

Benenson *et al*. [8] first described several security issues in WSNs, especially the access control problem, and proposed the notion of *n*-authentication, where users can successfully authenticate with at least (*n-t*) of n-sensors, where *t* is the number of sensor nodes that the adversary can compromise. Subsequently, Benenson *et al*. [9] proposed another solution for the user authentication problem in the face of node capture attacks. The proposed scheme is based on public key cryptography (*PKC*) and elliptic curve cryptography (*ECC*). Some major weaknesses were pointed out in the Benenson *et al*. scheme, such as the fact that impersonation attacks or denial-of-service (*DoS*) attacks could be mounted by sending many bogus signatures during the authentication phase [10]. Moreover, the computation cost of *PKC* and *ECC* is very high for sensor networks.

Wong *et al*. [10] proposed a dynamic user authentication scheme for wireless sensor networks, which is based on passwords. This scheme imposes a very light computation cost that requires only one-way hash functions and simple *XOR* operations. The scheme consists of three phases: registration phase, login and authentication phase. Unfortunately, the Wong *et al.* scheme is vulnerable to many attacks such as replay attacks, forgery attacks, stolen-verifier attacks and password guessing attacks [11,12,14,18].

Vaidya *et al*. [14] pointed out some weaknesses of the Tseng *et al*. [11], Wong *et al*. [10] and Ko [14] schemes such as replay of account-login attacks, man-in-the-middle (*MITM*) attacks, forgery attacks and stolen-verifier attack with node capture attacks. They proposed two user authentication schemes for wireless sensor networks, which are based on traditional password schemes and claimed that their proposed scheme provides better security features as compared to the Wong *et al*., Tseng *et al*. and Ko *et al*. schemes.

Recently, Das [18] pointed out some security flaws in Wong *et al*. [10] scheme, such as the fact this scheme is vulnerable to many logged in users with the same login-id threat and also susceptible to stolen-verifier attacks. Das [18] proposed a two-factor user authentication for wireless sensor networks, where the legitimate users must prove the possession of both a password and a smart card. Das [18] claimed that his scheme is secure against many types of attacks (e.g., *user authentication*, *replay*, *guessing*, *impersonation*, *node compromise*, and *stolen-verifier attacks*).

Nyang and Lee [19] noted that Das’ scheme is not practical and is vulnerable to an offline password guessing attack by insiders, node compromise attacks and does not care about other security issues, *i.e*., encryption and authenticity verification of query responses. Consequently they proposed an enhanced two-factor user authentication protocol for WSNs, which overcome the Das scheme’s security flaws with some additional security services such as confidentiality and authenticity of user query responses. However, their scheme also does not care about mutual authentication and there is no provision for password updates.

Khan and Alghathbar [20] pointed out that the Das *et al*. [18] scheme is still not secure and cannot resist many other security attacks, such as gateway-node bypass attacks, and it is vulnerable to insider attacks, as well as not facilitating mutual authentication between the gateway and the sensor nodes and there is no provision for users to change their passwords. Khan and Alghathbar [20] overcome the security weaknesses of Das’ scheme and proposed an improved two-factor user authentication in WSNs, which provides protection against insider attacks, gateway bypass attacks and introduced a password change phase for users. They suggested two secret (*X_a_* and *X_s_*) values to be used to overcome the gateway-bypass attacks. For example, *X_a_* is used between the user and the gateway, and *X_s_* is used between the gateway and the sensor nodes. Furthermore, to overcome the room for insider attack, they passed hashed passwords instead of plain passwords. Their scheme also does not however provide mutual authentication between the user and the gateway.

More recently, He *et al*. [21] have shown that Das’ protocol is susceptible to insider attacks, impersonation attacks and also found design weaknesses (*i.e.*, *concerning real identity of user*). Later, they proposed an enhanced two-factor user authentication scheme for WSNs that facilitates user anonymity, protection against insider attacks and allows users to change their passwords. Their scheme does not care about mutual authentication between all parties (*i.e.*, *user*, *gateway* and *sensor*). Moreover, the communication cost is quite high as compared with other schemes (see [10–14,18–20]).

As we have seen above, a number of user authentication schemes have been proposed in order to authenticate user legitimacy for sensor networks. These schemes are not designed properly [10–14] (*i.e.*, *stored secrets at the gateway*) and in [18–21] they do not provide essential services to the users. Consequently, we conclude that the above schemes have many security flaws and provide less security services. Thus, these security weaknesses and constrained nature of sensor nodes motivated us to design a robust user authentication framework that provides adequate security and provides users with many services, as discussed in the next section.

## 3. Design Goals, Security Requirements and System Architecture

In this section, we discuss the design goals, security requirements and system architecture for robust user authentication for wireless sensor networks.

### 3.1. Design Goals and Security Requirements

RUASN provides transparent security service for wireless sensor networks. In this paper another goal is to design a simple and user-friendly framework, which will be suitable for real-time WSNs applications. Overall, RUASN is designed with the following characteristics:

***Proper user authentication***: A user must prove his/her authenticity, so that only authentic users can access the WSN data.
***Mutual authentication***: Every entity (*user*, *gateway* and *sensor*) must be mutually authenticated; hence they can ensure the communication is only taking place between authentic entities.
***User anonymity***: Since *all* the messages are broadcasted wirelessly, which means that an attacker may simply eavesdrop on the messages and could breach the privacy of a user, thus, the scheme must provide user anonymity.
***Session key establishment:*** A *session* key should be established between a user and sensor node, so that subsequent communication could take place securely.
***Confidentiality:*** It is desirable that a user authentication protocol facilitate confidentiality of messages; as a result, these confidential messages can only be used by authorized users.
***Password update:*** A *password* based user authentication scheme should provide users a password update facility so that a user can update his/her password freely.
***Low communication and computational cost:*** Since sensor nodes are resource constrained devices (e.g., *MicaZ [25]*, and *Telosb [26]*) and, in general, application functions also need room for executing their tasks. So, the scheme must be efficient in terms of communication and computational cost.
***Robust against popular attacks:*** Clearly, the scheme should defend against different popular attacks, such as replay, impersonation, insider, stolen-verifier, password guessing, and man-in-the-middle attacks. As a result, the scheme should be easily applicable to real-world applications.
***User-Friendliness***: The system *architecture* should be easy to deploy for the WSN applications as well as user-friendly for non-advanced users so users can update his/her password securely according to their will.
***Reliability***: From the security *point* of view, the gateway needs to check the validity of the users and the sensor nodes, so that reliable communication can be established between all the parties.

Moreover, Liao *et al*. [27] identified some security requirements to evaluate a smart card and password based authentication protocol. Their requirements solve most of the problems in smart card oriented schemes. As sensor networks are resource constrained devices, WSN could adopt Liao *et al*. requirements, which are listed below:

The password tables should not be stored inside the gateway.
Verification table should not be stored inside the gateway.
The password should not be transmitted as plain text over the public network.
Schemes should resist insider-attacks.
Password length should be sufficient.
The password is not exposed by the gateway administrator.
Scheme should resist offline password guessing attacks.

### 3.2. System Architecture

**Figure 1.**
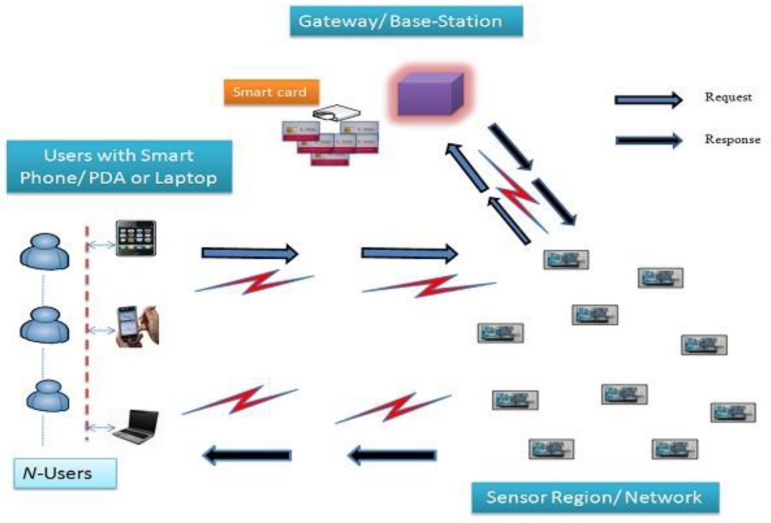
The basic system architecture for RUASN.

Wireless sensor networks consist of a number N of low-cost sensor devices, which are scattered in a hostile environment. These sensors sense the environmental information, (e.g., *humidity*, *pressure*, *and temperature*) and transmit information to the users for further analysis. A user can access real-time WSN data using their mobile devices (e.g., *laptop*, *PDA or smart-phone*) through wireless communication. WSN data could be easily accessible from anywhere in ad-hoc manner. In real-time environment it is obvious that the gateway nodes and the users are able to access the sensor data directly. The basic system architecture is shown in Figure 1, where a user directly request to the sensor node, upon receiving user request sensor node first verify user authenticity through the gateway node.

After confirmation of the user’s legitimacy he/she can access the real-time sensor data. Furthermore, the gateway node provides a middle ground between the users and the sensor network.

## 4. RUASN: Robust User Authentication for Wireless Sensor Network

To solve the potential problems of user authentication for WSNs, we propose RUASN which ensures WSN data are only accessed by legitimate users. Thus, before issuing a query to a sensor node, each user must register with the gateway in a secure manner so that they can access the real time sensors’ data. Upon the successful user registration request, the gateway node personalizes a smart card for every registered user, as shown in Figure 1. Then, a user can submit his/her query in an authentic way and access the sensor network data at any time within an administratively configurable period [10].

In order to execute the proposed framework, we considered that the gateway is a trusted node and it hold two master keys (*x and y*), which are sufficiently large for the sensor network. Before starting the system, it is assumed that the gateway and the sensor nodes share a long-term common secret key, *i.e*., *SK_gs_* *= h*(*Sn||y*) using any key agreement protocol. For example, [4] demonstrated that, with the careful design, D-H key agreement protocol [28] and RSA public key cryptosystem [29] can be easily deployed on most constrained devices. Here, *h*(.) is a collision free one-way hash function (*i.e.*, *SHA-1*), which has an output length of 160-bits [30] and is used throughout this paper. It is assumed that some identical secure symmetric cryptosystems are publically available and stored on the user device, on the gateway and the sensor node. As a result only the users registered with the gateway have access privileges to the sensors, which share a long-term secret with the gateway. The framework is divided into four phases, namely, user registration phase, login phase, authentication phase and password update phase. For convenience Table 1 provides a list of some notations and symbols will be used throughout the rest of paper.

### 4.1. Registration Phase (RP)

In the registration phase, initially, each user must register with the *GW* node. A user *U_k_* chooses his/her identity (*ID_k_*). Now user *U_k_* chooses password (*PW_k_*) and selects an arbitrary random number *r* which should be sufficiently large and computes *h*(*r ⊕ PW_k_*). The *ID_k_* and *PW_k_* should include uppercase, lower case characters and 0–9 numeric characters [32]. Afterward, the user submits a registration request to the *GW* node over a secure channel. Upon receiving the *U_k_* registration request, the *GW* node computes:

1. *A_k_=h(ID_k_⊕l)*
2. *B_k_= E_x_[ID_k_||l]*
3. *V_k_= h(ID_k_||h(r⊕PW_k_))*

**Figure 2.**
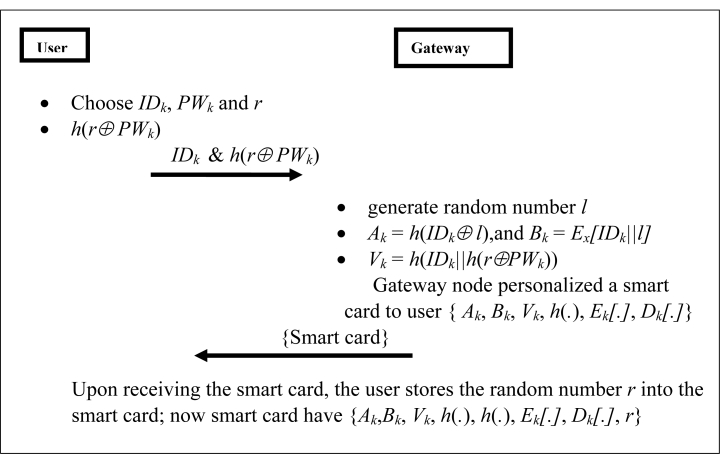
Flow of registration phase.

Thereafter, the *GW* node personalizes a smart card for the user with the parameters *{A_k_*, *B_k_*, *V_k_*, *h*(.), *E_k_[.]*, *D_k_[.]}*. Here, *l* is random number which is generated by the *GW* node and *x* is the gateway secret key and *h*(.) is a collision free one-way function, e.g., SHA-1 [30]. Subsequently, user *U_k_* enters *r* into his/her smart card, by doing so, user *U_k_* need not memorize the arbitrary random number. Now the smart card contains *{A_k_*, *B_k_*, *V_k_*, *h*(.), *E_k_[.]*, *D_k_[.]*, *r}*. This step completes the registration phase and the process flow is shown in Figure 2.

### 4.2. Login Phase (LP)

**Figure 3.**
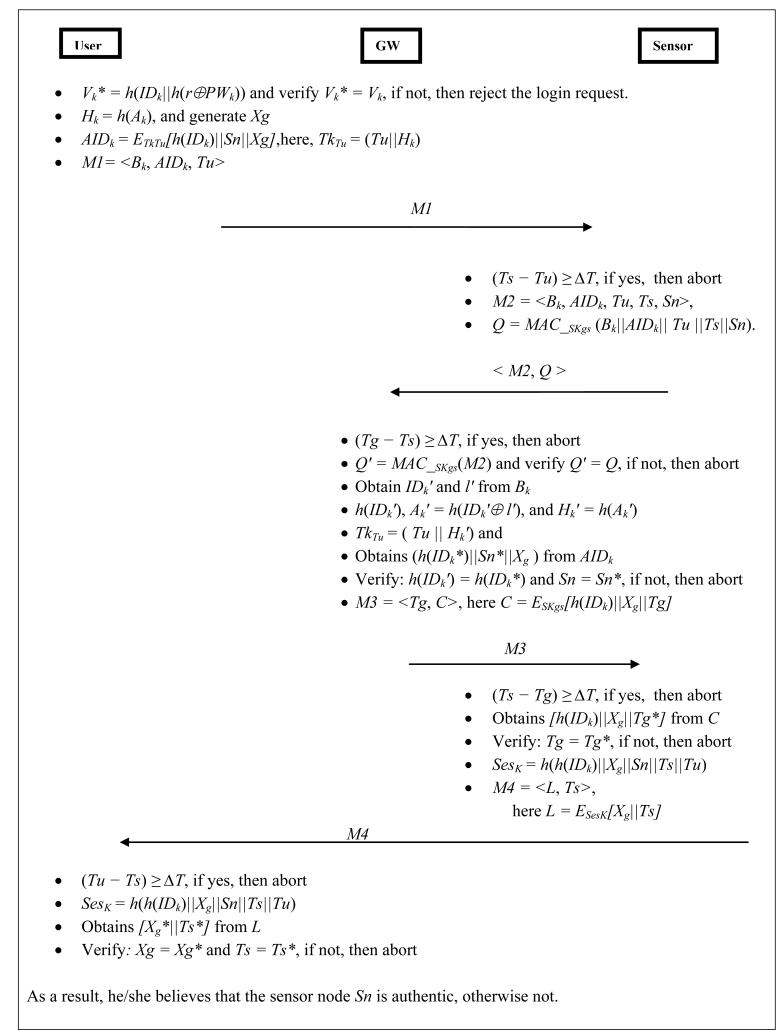
Flow of login and authentication phases.

This phase is invoked whenever a user *U_k_* wants to submit his/her query to access the sensor, every time he/she has to complete the login phase. Figure 3 shows both the login phase and the authentication phase. The user *U_k_* inserts his/her smart card into the terminal and inputs his *ID_k_* and *PW_k_*.

Now, the smart card performs the following operations:

(LP-1). Compute: $V_{k}^{*}\;=\;h(ID_{k}||h(r⊕PW_{k}))$.
(LP-2). Check whether $V_{k}^{*}$ and *V_k_* are equal or not. If not, then reject the login request, otherwise, the user is a legal user and go to the next step.
(LP-3). Compute: *H_k_* *= h*(*A_k_*).
(LP-4). Compute: *AID_k_* *= E_TkTu_[h*(*ID_k_*)||*Sn||X_g_]*; here, *Tk_Tu_* *=* (*Tu||H_k_*) is short-term key and *Sn* is the sensor node, which the user *U_k_* wants to access. Here, *X_g_* is a secret random number generated by the user *U_k_* at login time, which is helpful to generate the session key between the user and the sensor node. *Tu* is denoting the current timestamp of *U_k_*’s system, which resist replay attacks.
(LP-5). Send the login message *M1 = < B_k_*, *AID_k_*, *Tu >* to the sensor node.

Now, this is the end of login phase and the user login request message <*M1>* is send to the sensor node over a public channel.

### 4.3. Authentication Phase (AP)

The authentication phase is invoked when a sensor node receives the user login request message <*M1>* at time *Ts*. The sensor node authenticates users’ requests by the following steps:

(AP-1). The sensor node, *Sn* validates the *Tu*: Check, if (*Ts–Tu*) ≥ Δ*T*, if yes, then *Sn* reject this request and terminates the process. Otherwise, it continues with the next step. Here, *Ts* is the current timestamp of *Sn* and Δ*T* is the defined time interval for the transmission delay.
(AP-2). Now *Sn* generates message *M2 = <B_k_*, *AID_k_*, *Tu*, *Ts*, *Sn>* and *Q*. Thereafter, *Sn* sends <*M2*, *Q>* to the *GW* node over public network. Here, a message authentication code (*MAC*) [3,33] is computed on the message *M2* (*i.e.*, *Q = MAC__SKgs_*((*B_k_||AID_k_||Tu||Ts||Sn*))) for the integrity verification by the *GW* node.
(AP-3). Upon receiving the message *M2* and *Q* from the sensor node, the *GW* node performs the following actions:
  1. The *GW* node validates the time *Ts*: Check if (*Tg–Ts*) ≥ Δ*T*, if yes, then the *GW* node rejects this request and terminates the process, otherwise, it continues with the next step. Here, *Tg* is the current timestamp of the *GW* node and Δ*T* is the defined time interval for the transmission delay.
  2. The *GW* node computes *Q′* on the message *M2* using long-term secret key *SK_gs_* and verifies *Q′ = Q*, if yes, then the *GW* node considers that this is an original message and proceeds to the next step, otherwise, it rejects the request and terminates further operations.
  3. The *GW* node decrypts *B_k_* using secret key *x* and obtains $ID_{k}^{'}$ and *l′* of *U_k_*. Now, the *GW* node computes $h(ID_{k}^{'})$, $A_{k}^{'}\;=\;h(ID_{k}^{'}⊕l')$ and $H_{k}^{'}\;=\;h(A_{k}^{'})$. Subsequently, the *GW* node generates a temporary key *Tk_Tu_* (*Tu||* $H_{k}^{'}$). After that, the *GW* node decrypts the sub-message *AID_k_* by using the *Tk_Tu_* and obtains *[h*( $ID_{k}^{*}$)||Sn*||X_g_]. Afterwards, the *GW* node compares $h(ID_{k}^{'})$ with $h(ID_{k}^{*})$, if this check is successful then the user is a legal user. Here, $h(ID_{k}^{*})$ is sub-message of *AID_k_*. At the same time, the *GW* node verifies whether *Sn* is equal to Sn*, which is included in sub-message of *AID_k_* and if yes, then the *GW* node considers that *Sn* is a legal node that user *U_k_* wants to access. Otherwise, the *GW* node rejects the authentication process.
  4. After authenticating the user and the sensor node; the *GW* node informs the sensor node that user *U_k_* is a legitimate user, therefore, the *GW* node computes message *M3 = <Tg*, *C>* and sends message *M3* to the sensor node. Here, *C = E_SKgs_[h(ID_k_)||X_g_||Tg]* and *Tg* is the current timestamp of the *GW* node.
(AP-4). Upon receiving the message *M3* from the *GW* node, the sensor node performs the following:
  1. Firstly, *Sn* validate the time *Tg*: Check if (*Ts–Tg*) ≥ Δ*T*, if yes, then the sensor node rejects this request and terminates the process. Otherwise, it continues with the next step. Here, *Ts* is the current timestamp of the sensor node and Δ*T* is the defined time interval for the transmission delay.
  2. Now, with the knowledge of *SK_gs_*, the sensor node decrypts the sub-message C = D_SKgs_[h(ID_k_)||Xg||Tg*] and verifies whether *Tg* is equal to Tg* or not. If *Tg* is not verified then the process is terminated; otherwise, the sensor node considers the *GW* node a legal node and message *M3* is generated by the original *GW* node.
  3. Thereafter, *Sn* computes a session key *Ses_K_* *= h*(*h*(*ID_k_*)*||Xg||Sn||Ts||Tu*) for secure communication between the sensor node and the user. Now, *Sn* generates a message *M4 = <L, Ts>*, and sends it to the user *U_k_*. Here, *L = E_SesK_[Xg||Ts]* and *Ts* is the current time stamp of the sensor node.
(AP-5). While receiving the message *M4* from the sensor node, the user *U_k_* performs the following:
  1. Firstly, *U_k_* validates the time *Ts*: Check if (*Tu – Ts*) ≥ Δ*T*, if yes, then the sensor node rejects this request and terminates the process. Otherwise, it continues with the next step. Here, *Tu* is the current timestamp of the user *U_k_* and Δ*T* is the defined time interval for the transmission delay.
  2. Now, the user *U_k_* computes the session key *Ses_K_* *= h*(*h(ID_k_*)*||X_g_||Sn||Ts||Tu*) and decrypts the sub-message *L* (*i.e.,* $D_{SesK}[X_{g}^{*}\left|\right|Ts*]$) and obtains $X_{g}^{*}$ and Ts*. The user *U_k_* checks $X_{g}\;=\;X_{g}^{*}$ and Ts = Ts*, if yes, then he/she believes that *Sn* is a real sensor node, otherwise not.
  3. Moreover, now the user *U_k_* and *Sn* share the symmetric session key *Ses_K_* *= h*(*h*(*ID_k_*)*||X_g_||Sn||Ts||Tu*) for performing further subsequent operation during a session.

As a result, a legitimate user can communicate with real sensor nodes and access the network data.

### 4.4. Password-Update Phase (PUP)

The password update phase is invoked whenever user *U_k_* wants to update his/her old password (*PW_k_*). The password update phase is described below:

(PUP-1). User *U_k_* inserts his/her smart card into the terminal and enters his/her identity (*ID_k_*) and password (*PW_k_*).
(PUP-2). Firstly, the smart card validates the *U_k_*’s entered *ID_k_* and *PW_k_* with the stored values by computing the following: Vk* = h(*ID_k_||h*(*r⊕PW_k_*)).
(PUP-3). Verify whether $V_{k}^{*}$ and *V_k_* are equal or not. If not, then reject the password update request; otherwise, proceed to the next steps.
(PUP-4). Upon receiving the *U_k_’s* new password ( $PW_{knew}^{*}$), the smart card computes $V_{knew}^{*}\;=\;h(ID_{k}||h(r⊕PW_{knew}^{*}))$ (PUP-5). Now, the smart card replaces *V_k_* with $V_{knew}^{*}$.

**Figure 4.**
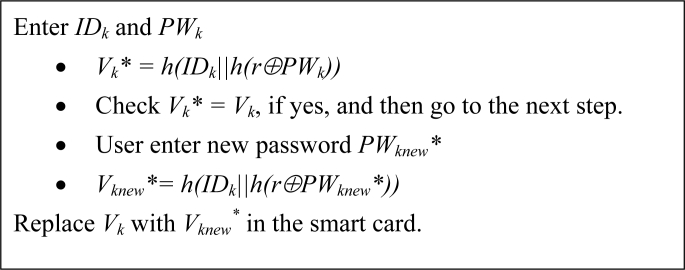
Flow of password update phase.

After performing the above steps, the password update phase takes place successfully. The flow of the password update phase is shown in Figure 4.

## 5. Nonmonotonic Cryptographic Protocol and RUASN Analysis and Verification

This section presents, first, the nonmonotonic cryptographic protocol (*NCP*) [24], and second, formal analysis and verification of *RUASN* using the *NCP* protocol.

### 5.1. Nonmonotonic Cryptographic Protocol

This sub-section describes the nonmonotonic cryptographic protocol (*NCP*), which is also known as Rubin Logic. The *NCP* logic assumed that involved entities are trusted, and state of knowledge (*i.e., current state of the protocol*), and describes the authentication logic. In [24–34] the authors state that the *NCP* logic does not require any idealization step in specifying the protocol and is very close to real implementation. In Rubin logic the roles are assigned to entities, which are considered as independent processes. The nonmonotonic logic consists of two sets, global set and local set. These sets are directly applicable for protocol analysis, as follows. For more details the reader may refer to [24] and [35].

#### 5.1.1. Global Set and Local Set

*Global set:* The global sets are publically known to each principal in a protocol specification, and consist of principal set, rule set, secret set and observers set. These sets represent the information of the protocol and the contents of these global sets may get updated as the protocol progresses.

- ➢ *Principal set:* This set represents the involved entities in a protocol, such as, *Et = {Et_1_*, *Et_2_*, *Et_3_*,*…..*, *Et_n_}*.
- ➢ *Rule set:* This set contain inference rules for deriving new statements from existing statements, as described in Section 5.1.3.
- ➢ *Secret set:* This set holds all the protocol secrets, such as *S = {S_1_, S_2_, S_3_,…., S_n_}*, that exist at any given time in the system.
- ➢ *Observers set:* This set holds all *Si* such that for each *Si*, Observers (*Si*) contains all the involved entities who could possibly know the secret *Si* in the system.
- ➢ *Local set:* The local sets are not publically known, instead, they are private to each entity. The local sets consist of the following:
- ➢ *Possession set* (*Ei*): This set holds all the data relevant to security, and that particular entity knows or possesses, which includes encryption keys, public keys, and other secrets that are not publically available. *POSS*(*Ei*) *= {poss_1_*, *poss_2_*, *psos_3_*,*….*, *poss_n_}*.
- ➢ *Belief set:* This set holds all the beliefs held by a principal. For example, the beliefs about freshness, and the beliefs about the possessions of other involved principals. *BEL*(*Ei*) *= {belf_1_*, *belf_2_*, *belf_3_*,*….*, *belf_n_}*.
- ➢ *Seen set* (*Ei*): This set holds plaintext message parts that *Ei* sees from messages sent across the network and it also contain a copy of the information as the Observers sets.
- ➢ *Behavior list* (*Ei*): This is a list instead of a set, and the list elements are ordered. *BL = {AL*, *bev_1_*, *bev_2_*, *bev_3_*,*…*, *bev_n_}*, here *AL* is an action list, which consists of zero or many actions executed by *Ei* and *bev_n_* is a pair, *i.e*., (*message*, *AL*). The messages have two forms: *Send* (*Ei*, *message*) and *Receive* (*Ei*, *message*). Furthermore, after every *Send*(.) operation, the Observers set has to be updated using *Update*(.) operation. After each *Update*(.) operation the control pass to the next *Receive*(.) operation of principal, which is specified in the earlier *Send*(.) operation.
- ➢ *Haskeys set* (*Ei*): The Haskeys set holds keys that *Ei* sees either because they are in the initial possession set, or they appear in a message sent across the network and are added to *Ei’s Seen set*.

#### 5.1.2. Actions

Actions are operations which play essential roles in the protocol specification. These actions control the state of knowledge and possessions for involved entities (*such as, constructs messages, hashing, concatenation, encryption /decryption, secret generation, update, and abort operations, are few example*). For a complete list of actions readers may refer to [24,35]. As per our requirements, we have defined the following actions, as shown below, which are directly adopted from [24–34]. The action is marked with ▪ to show that it has been successful and moved to the next action.

▪ *Hash* (*h*(.); *X*)
  Condition: *h*(.), *X ∈ POSS*(*Ei*)
  Result: *POSS*(*Ei*)*: = POSS*(*Ei*) *∪ {h*(*X*)*}*
  Description: This action is for hashing the data.
▪ *XOR*(*X_1_*, *X_2_*, *X_3_*, ….., *X_n_*)
Condition: *X_1_, X_2_, X_3_, ….., X_n_* *∈ POSS*(*Ei*)
  Result: *POSS*(*Ei*): = *POSS*(*Ei*) *∈ {X_1_, X_2_, X_3_, ……,X_n_}*
  Description: This action is for *XOR*ing the data.
▪ *Encrypt*(*X*, *k*)
  Condition: *X*, *k* ∈ *POSS*(*Ei*)
Result: *POSS*(*Ei*) : = *POSS*(*Ei*) *∪{{X}_k_}*
  Description: This action occurs when a principal encrypts data. If *Ei* possesses *X* and knows *k* then he/she can possess *{X}_k_*.
▪ *Decrypt*(*{X}_k_*, *k*)
  Condition: *{X}_k_*, *k* ∈ *POSS*(*Ei*)
  Result: *POSS*(*Ei*):= *POSS*(*Ei*) *∪* {*X*}
Description: This action is performed when a principal decrypts data. If *Ei* possesses *X*, encrypted under *k*, and *Ei* knows *k*, then he/she (*Ei*) can possess *X*.
▪ *Generate-Secret*(*X_g_*)
Result: *S:= S ∪ {X_g_}, Observers*(*X_g_*) *= {Ei}*, *POSS*(*Ei*) *:= POSS*(*Ei*) *∪* {*X_g_*, *Ei}*, *BEL*(*Ei*):= *BEL* (*Ei*) *∪* #(*X_g_*)
Description: This action generates a secret for an entity, when needed. Thereafter, a new secret *X_g_*, is added to secret *S* and the Observers and Possession sets are updated.
▪ *Concat*(*X_1_, X_2_, X_3_, ….,X_n_*)
  Condition: *X_1_, X_2_, X_3_, …..,X_n_* *∈ POSS*(*Ei*)
  Result: *POSS*(*Ei*) := *POSS*(*Ei*) *∪* {*X_1_, X_2_, X_3_, …., X_n_}*
  Description: This action concatenates the sub-messages.
▪ *Check*(*X*, *Y*)
  Condition: *X*, *Y ∈ POSS*(*Ei*)
  Result: Valid if *X==Y*, otherwise invalid.
▪ *Split*(*X*)
Condition: *X contains X_1_, X_2_, X_3_, ….., X_n_, X* ∈ *POSS*(*Ei*)
  Result: *POSS*(*Ei*):= *POSS*(*Ei*) *∪ {X_1_, X_2_, X_3_,….,X_n_}*
  Description: This action is used to break the message into sub-messages.
▪ *Send*(*Ej*, *X*)
  Description: This action sends message *X to Ej* (*i.e.*, *Ei to Ej*).
▪ *Receive*(*Ej*, *X*)
  Description: This action receives message *X* from *Ej* (*i.e.*, *Ei receives from Ej*) and added to *POSS*(*Ei*).
▪ *Update*(*X*)
  Description: The purpose of update action is to update the Observers sets of all secrets that are sent on the network.
▪ *Forget*(*X*)
  Description: The purpose of forget action, when *Ei* no longer is in possession of *X*.
▪ *Abort:*
  Description: The abort action takes place when a checked action could not satisfy the established conditions. Furthermore, this action aborts the system, if a protocol run is illegal. As a result, analysis reports a failure.

#### 5.1.3. Inference Rules

A procedure which combines known facts and produce new fact is called an inference rule [24]. These inference rules are used to fact about beliefs during the protocol execution and are applied whenever they are relevant to protocol progress. The following inference rules are defined as per our requirements, which are directly adopted from [24,35].

**Notation:**
***X contains Y:*** *Y* appears as a sub-message of *X*.
***S: = f*(*S*)**: *S* is replaced by the value of *f*(*S*).
***X from E:*** *X* is received from *E*.

1. *Message-meaning rule:*
  $$\frac{{\left\{X\right\}}_{k}\;\text{from}\;Ej\in \;\text{POSS}(Ei),\;\{Ei,\;Ej\}\;\subseteq \text{POSS}(Ei)}{\text{BEL}\;(Ei):\;=\;\text{BEL}\;(Ei)\;\cup \{X\;\in \;\text{POSS}\;(Ei)\}}$$
2. *Origin rule:*
  $$\frac{X\;\in \;\text{POSS}(Ei),\;X\;\text{contains}\;x_{1},\;Ej\;\in \;\text{Observers}(x_{1})}{x_{1}\;\text{from}\;Ej\;\in \;\text{POSS}\;(Ei)}$$
3. *Sub-message origin rule:*
  $$\frac{X\;\in \;\text{POSS}\;(Ei),\;X\;\text{contains}\{x_{1},\;x_{2}\}\;\text{from}\;Ej}{x_{2}\;\text{from}\;Ej\;\in \;\text{POSS}(Ei)}$$
4. *Sub-message freshness rule:*
  $$\frac{\#(x_{1})\;\in \;\text{BEL}\;(Ei),\;\{X\;\text{contains}\;x_{1},\;Y\;\text{contains}\;x_{2}\}\;\subseteq \;\text{POSS}\;(Ei)}{\text{BEL}\;(Ei):\;=\;\text{BEL}(Ei)\;\cup \;\#(x1)}$$

For a complete list of inference rules, readers may refer to [24] and [35].

### 5.2. RUASN Analysis and Verification Using Rubin-Logic

This sub-section presents the analysis and verification of the proposed *RUASN* using well-known Rubin logic [24]. The *NCP* logic integrates protocol analysis with protocol specification and thus, beliefs of involved entities and current state of knowledge is updated as the protocol run progresses. For *RUASN* analysis and verification, only three phases are accounted, namely, registration phase, login phase and authentication phase, as follows.

#### 5.2.1. RUASN Specification

For convenience, a list of some additional notations and symbols will be used in the *RUASN* analysis and verification, as shown in Table 2.

As per the *NCP* logic, the specifications of *RUASN* are the following:

*Global sets:* It consists of four sets:

▪ *Principal set: P = U*, *GW*, and *Sn*. Here, *U* is the initiator of *RUASN*.
▪ *Rule set:* Inference rules are defined in Section 5.1.3.
▪ *Secret set: {PW_k_, r, x, y, l, SK_gs_}*
▪ *Observers set:*
  *Observers* (*PW_k_,r*)*: {U}*
  *Observers*(*x*, *y*, *l*)*: {GW}*
  *Observers* (*SK_gs_*)*: {GW, Sn}*

*Local sets:* The local sets consist of *U*, *GW*, and *Sn* and are as shown in Table 3.

#### 5.2.2. RUASN Analysis and Verification

Once the protocol specification has been completed, the analysis begins. This subsection analyzes the proposed scheme, which is very close to real implementation. We have considered three phases, namely, registration phase, login phase and authentication phase, where three entities are involved in the protocol progress [*i.e.*, *user*(*U*), *gateway*(*GW*) and *sensor*(*Sn*)].

As we can see the Phase-I in Table 3, the entity *U* is the initiator of the protocol, so user behavior list actions are executed first [*i.e.*, *BL*(*U*)]. The first three actions are executed in *BL*(*U*) and once the *Update* action is performed, the next actions have to be executed in the *GW* behavior list, since the *Send* operation (*i.e.*, *Send*(*GW*,*{ID_k_*,*Pass}*)) is specifies *GW* list, as shown below:

▪ *Hash*(*h*(.)*; XOR*(*r*, *PW_k_*))→*Pass*
▪ *Send*(*GW*, *{ID_k_, Pass}*)
▪ *Update*(*{ID_k_, PW_k_, r}*)

Now GW’s Phase-I actions behavior lists takes place [*i.e.*, *BL*(*GW*)] and first seven actions are executed in *BL*(*GW*). After the *Update* operation, the next operations have to be executed in the user’s behavior list as below, the *Send* operation [*Send*(*U*, *{A_k_*, *B_k_*, *V_k_*, *h*(.),*E_k_,D_k_}*)] assigned to *U*. Then the *Forget* operation removes all values (*i.e.*, *A_k_*,*B_k_*,*V_k_,E_k_,D_k_*) from the local possession set [*POSS*(*GW*)], as shown below:

▪ *Receive*(*U*, *{ID_k_, Pass}*)
▪ *Generate-secret number*(*l*)
▪ *Hash*(*h*(.)*; XOR*(*ID_k_, l*))→*A_k_*
▪ *Encrypt*(*{Concat*(*ID_k_, l*)*}_x_)→B_k_*
▪ *Hash*(*h*(.)*; Concat*(*ID_k_*, *Pass*))*→V_k_*
▪ *Send*(*U*, {*A_k_,B_k_,V_k_, h*(.),*E_k_,D_k_}*)
▪ *Update*(*{A_k_, B_k_, V_k_, h*(.),*E_k_,D_k_, Pass}*)
▪ *Forget*(*{A_k_, B_k_, V_k_, E_k_,D_k_, Pass}*)

It is clear that after the end of the user and the gateway Phase-I, there is no significant change in their global sets, whereas, in the local sets of both entities (*i.e*., *user* and *gateway*) have are some changes, as shown:

*POSS*(*U*) = *{ID_k_, PW_k_, r{A_k_, B_k_, V_k_, h*(.),*E_k_,D_k_}}*
*BEL*(*U*) = *{#*(*PW_k_*)*}*
*POSS*(*GW*) = *{x*,*y*,*l*,*SK_gs_}*
*BEL*(*GW*) = *{#*(*x*), *#*(*y*), *#*(*l*), *#*(*SK_gs_*)*}*

This is the end of Phase-I.

Subsequently, Phase-II begins and two entities (*i.e.*, *User* and *Sensor*) are involved in this phase. As, *U* is the initiator of the protocol, and thus next operations have to be executed in *U’s* behavior list (*BL*(*U*)), as follows:

▪ *Hash*(*h*(.)*; Concat*(*ID_k_, Pass*))→ $V_{k}^{*}$
▪ *Check*( $V_{k}^{*}$, *V_k_*)
▪ *Hash*(*h*(.)*; A_k_*)→*H_k_*
▪ *Generate-secret*(*X_g_*)
▪ *Concat*(*Tu*, *H_k_)→TkTu*
▪ *Encrypt*(*{Concat*(*Hash*(*h*(.)*;ID_k_*, *Sn*, *X_g_*))*}_TkTu_*)→*AID_k_*
▪ *Send*(*Sn*,*{B_k_, AID_k_, Tu}*)→*M1*
▪ *Update*(*ID_k_, X_g_, Sn*, *Tu*)

After the *Update* operation, the following changes will takes place in *BL*(*U*).

*POSS*(*U) = {ID_k_*, *V_k_*, *X_g_*, *TkTu*, *AID_k_*, *A_k_*, *H_k_}*
*BEL*(*U*) = *{#*(*X_g_*), *#*(*TkTu*)*}*

The global set of entity *U* will change with the following:

- *Observers*(*X_g_*)*: {U}*

The *next* operations have to be executed in *Sn’s* behavior list [*BL*(*Sn*)], because the above *Send* operation (*i.e.*, *Send*(*Sn*,*{Bk*, *AIDk*, *Tu}*)*→M1*) specifies *Sn*. Upon receiving the *Receive* operation (*i.e.*, *Receive* (*U*,*{M1}*)), *Sn* does the following:

▪ *Receive*(*U*, *{M1}*) *[*Here *M1 =* (*B_k_, AID_k_, Tu)]*
▪ *Split*(*M1*)
▪ *Check-freshness* (*Ts* – *Tu*) ≥Δ*T*, if yes, then aborts.
▪ *MAC*(*{Concat*(*B_k_*, *AID_k_*, *Tu*, *Ts*, *Sn*)*}SK_gs_*)*→Q*
▪ *Send*(*GW*, *{M2*, *Q}*) *[*Here *M2* = (*B_k_,AID_k_, Tu*, *Ts, Sn*)*]*
▪ *Update*(*M2*, *Q*)

The *Update* operation makes the following changes in *BL*(*Sn*):

- *POSS*(*Sn*) *= {B_k_*, *AID_k_*, *Tu*, *Ts*, *Sn*, *Q*, *SK_gs_}*
- *BEL*(*Sn*) *= {#*(*SK_gs_*)*}*

As we can see the next operations will be executed in *GW’s* behavior list [*BL*(*GW*)], as the above *Sn Send* operation (*i.e.*, *Send*(*GW*,*{M2*, *Q}*)) specifies to *GW*. Upon receiving the *Receive* operation [*i.e.*, *Receive* (*Sn*,*{M2*,*Q}*) ] from *Sn*. Now, *BL*(*GW*) performs the following operations:

▪ *Receive*(*Sn*, *{M2*, *Q}*)*[*Here *M2 = B_k_*, *AID_k_*, *Tu*, *Ts*, *Sn]*
▪ *Split*(*M2*, *Q*)
▪ *Split*(*M2*)
▪ *Check-freshness* (*Tg* – *Ts*) *≥* Δ*T*, if yes, then aborts.
▪ *MAC*(*{B_k_*, *AID_k_*, *Tu*, *Ts*, *Sn}_SKgs_*)*→Q′*
▪ *Check*(*Q*, *Q′*)
▪ *Decrypt*(*{B_k_}_x_*) and obtain *[* $ID_{k}^{'}$, *l′]*
▪ *Hash*(*h*(.); $ID_{k}^{'}$)
▪ *Hash*(*h*(.)*; XOR*( $ID_{k}^{'}$, *l′*)) *→A_k_′*
▪ *Hash* $h(.);A_{k}^{'})\to H_{k}^{'}$
▪ *Concat*(*Tu*, $H_{k}^{'}$)*→TkTu*
▪ *Decrypt*(*{AID_k_}_TkTu_*) and obtain*[h*( $ID_{k}^{*}$), Sn*, *X_g_]*
▪ *Check*(*h*( $ID_{k}^{*}$), *h*( $ID_{k}^{'}$))
▪ *Check*(Sn*, *Sn*)
▪ *Encrypt*(*{Concat*(*Hash*(*h*(.)*; ID_k_*), *X_g_*, *Tg*)*}_SKgs_*)*→C*
▪ *Send*(*Sn*, *{ Tg*, *C }*)*→M3*
▪ *Update*(*Tg*, *C*)

After applying *Update* operation the following changes take place in the gateway entity:

*POSS*(*GW*) *= {ID_k_*, *H_k_*, *X_g_*, *SK_gs_*, *Sn*, *l*, *x*, *y*, *{B_k_*, *AID_k_*, *Tu*, *Ts*, *Sn}* and *{MAC{B_k_*, *AID_k_*, *Tu*, *Ts*, *Sn}_Skgs_}*from *Sn}*
*BEL*(*GW*) *= {#*(*SK_gs_*),*#*(*x*), *#*(*y*), *#*(*l*),*#*(*Tg*)*}*

Subsequently, the global set of entity *GW* will change with the following:

*Observers*(*X_g_*)*: {U*, *GW}*

The next operations have to be executed in Phase-III of *BL*(*Sn*), because the above *Send* operation [*i.e.*, *Send*(*Sn*, *{Tg*, *C}*)] specifies *Sn* and upon receiving the *Receive* operation [*i.e.*, *Receive*(*GW*,*{Tg*, *C}*)] *Sn* will do the following:

▪ *Receive*(*GW*, *{Tg*, *C}*).
▪ *Split*(*Tg*, *C*).
▪ *Check-freshness* (*Ts* – *Tg*) *≥* Δ*T*, if yes, then aborts.
▪ *Decrypt*(*{C}_SKgs_*) and obtain *[h*(*ID_k_*, *X_g_*, Tg*)*].*
▪ *Check*(Tg*, *Tg*).
▪ *Hash*(*h*(.)*; Concat*(*Hash*(.)*; ID_k_*, *X_g_*, *Sn*, *Ts*, *Tu*))*→Ses_K._*
▪ *Encrypt*(*{Concat*(*X_g_*, *Ts*)*}_SesK_*)*→L.*
▪ *Send*(*U*, *{L*, *Ts}*)*→M4.*
▪ *Update*(*L*, *Ts*).

After applying the *Update* operation on the received message, the following changes will occur in the *Sn* entity:

*POSS*(*Sn*) *= { SK_gs_*, *Ses_K_*, *Ts{ID_k_*, *X_g_*, *Tg}SK_gs_* from *GW}*
*BEL*(*Sn*) *= {#*(*SK_gs_*), *#*(*Ses_K_*), *#*(*X_g_*), *#*(*Ts*)*}*

The following changes will occur in the global set of the entity *Sn*:

*Observers*(*X_g_*)*: {U*, *GW*, *Sn}*
*Observers*(*Ses_K_*)*: {Sn}*

Thereafter, the next operations have to be executed in Phase-III of *BL*(*U*). Since the above *Send* operation [*i.e.*, *Send*(*U*, *{L*, *Ts}*)] specifies *U* entity and *BL*(*U*) perform the following actions:

▪ *Receive*(*Sn*, *{L*, *Ts}*)*→M4 [*Here *L=ESes_K_[X_g_||Ts]]*
▪ *Split*(*{L*, *Ts}*)
▪ *Check-freshness* (*Tu* – *Ts*) *≥* Δ*T*, if yes, then aborts.
▪ *Hash*(*h*(.)*; Concat*(*Hash*(*h*(.)*; ID_k_*, *X_g_*, *Sn*, *Ts*, *Tu*)))*→Ses_K_*
▪ *Decrypt*(*{L}_SesK_*) and obtain ( $X_{g}^{*}$, Ts*)
▪ *Check*(*X_g_*, $X_{g}^{*}$)
▪ *Check*(*Ts*, Ts*)

In the *Check* operation *U*’s verify *X_g_*, which was generated by him/her and verify time-stamp *Ts*. If the *Check* operations are successful then the following changes will occur in *BL*(*U*):

Local set:

*POSS*(*U*) *= {X_g_*, *Sn*, *Ses_K_}*
*BEL*(*U*) *= {#*(*X_g_*), *#*(*Ses_K_*)*}*

Now finally the global set contains:

*Observers*(*X_g_*)*: {U*, *GW*, *Sn}*
*Observers*(*Ses_K_*)*: {U*, *Sn}*

The application of Rubin logic in our framework is illustrated above, which closely resembles the structure of real user authentication system in wireless sensor network. Our specifications are designed to resemble actual implementation as much as possible.

## 6. Evaluation of RUASN

In this section, we present our proposed *RUASN* evaluation in terms of security analysis (*i.e.*, *can it resist against several well-known attacks*), and efficiency analysis in terms of computational and communication cost. Finally, we show a functionality comparison with existing schemes.

Before evaluating the RUASN, it is assumed that an adversary may have full control over the network with following capabilities:

- An adversary may intercept all the messages (*i.e.*, *M1*, *M2*, *M3 and M4*) at any time.
- He/she may intercept, delete or modify, and insert any message over the public network.
- In addition, we assume that an adversary may hack either passwords or steal user *U_k_’s* smart card, extract secrets [36,37], but cannot do both at the same time [38].
- As per the current literature, extracting secrets from the smart card memory is quite difficult and some smart card manufacturer companies provide countermeasures against risk of side channel attacks [18,36]. Furthermore, [39] has proposed countermeasures against power analysis attacks.

Based on above assumptions, an attacker may execute certain attacks to breach the proposed RUASN scheme.

### 6.1. Security Analysis

In this subsection, we analyze the security of proposed RUASN and further compare with the M.L, Das [18], He *et al*. [21], Wong *et al*. [10], and Vaidya *et al*. [14] schemes. We prove that the presented scheme can resist certain popular attacks that are found in the existing wireless sensor network literature.

***Mutual Authentication***. Our scheme provides mutual authentication, where all entities (*i.e.*, *user*, *gateway* and *sensor node*) are mutually authenticating each other. More specifically, when the *GW* node receives the message *M2* (*i.e.*, *<B_k_*, *AID_k_*, *Tu*, *Ts*, *Sn>*) and *Q*, it can make sure that the user message *M1* (*i.e.*, *<B_k_*, *AID_k_*, *Tu>*) is included in the sensor node message *M2*. When the sensor node receives message *M3* (*i.e.*, *<Tg*, *C>*), it ensures that this message is generated by the *GW* node. Furthermore, when the user receives message *M4* (*i.e.*, *<L*, *Ts>*), he/she can also confirm that this message is generated by the sensor node. Hence, mutual authentication is achieved.

***User anonymity***. In our scheme, user anonymity *U_k_* is preserved at the registration phase by computing *A_k_* *= h*(*ID_k_⊕ l*) *and B_k_* *= E_x_[ID_k_||l]*. In addition, it is impossible to extract *ID_k_* from the *AID_k_*, which is *E_TkTu_[h*(*ID_k_*)*||Sn||X_g_]*, and it is also very difficult to revert the *h*(*ID_k_*). So, our scheme can preserve user anonymity.

***Session Key Establishment***. The proposed scheme provides session key establishment after the authentication phase. A session key [*i.e*., *Ses_K_* *= h*(*h*(*ID_k_*)*||X_g_||Sn||Ts||Tu*)] is set up between the user and the sensor node for secure subsequent communication. The *Ses_K_* will be different for each login session and cannot be replayed after the time expires. More importantly, the user and the sensor node can securely execute encryptions and decryptions by using of *Ses_K_* and hence, achieve confidentiality for the subsequent messages.

***Confidentiality***. Our proposed scheme provides adequate confidentiality to their messages (*such as*, *E_TkTu_[h*(*ID_k_*)*||Sn||X_g_]*, *E_SKgs_[h*(*ID_k_*)*||X_g_||Tg]* and *E_SesK_[X_g_||Ts]*). More precisely, these messages are confidential from any attacker.

***Replay Attacks***. Our scheme is resistant to replay attacks [42], because the authenticity of messages *<M1>*, *<M2>*, *<M3> and <M4>* are validated by checking the freshness of four timestamps ((*Ts – Tu*) *≥* Δ*T*, (*Tg* – *Ts*) *≥* Δ*T*, (*Ts* – *Tg*) *≥* Δ*T* and (*Tu* – *Ts*) *≥* Δ*T*). Let’s assume an intruder intercepts a login request message *M1* and attempt to access the sensor node by replaying the same message (*M1*). The verification of this login attempt fails, since the time difference expires (*i.e.*, (*Ts – Tu*) ≥ ΔT). Similarly, if an intruder intercepts a valid message *M2* (*i.e.*, *<B_k_*, *AID_k_*, *Tu*, *Ts*, *Sn>*) and attempts to replay it to the *GW* node, the verification request will fail at the *GW* node because of the time difference expires again (*i.e.*, (*Tg* – *Ts*) *≥* Δ*T*). Thus, our framework is secure against replaying of messages.

***User Impersonation Attacks.*** An attacker cannot impersonate the user. Suppose an attacker forges a login message *<B_k_*, *AID_k_*, *Tu>*. Now, he/she will again try to login into the system with the modified message < $B_{k}^{*}$, ${\text{AID}}_{k}^{*}$, *Tu*>, since, the fake ${\text{AID}}_{k}^{*}$ will not be verified at the *GW* node, and the *GW* node cannot get the original sub-message $\left\{h({\text{ID}}_{k}^{*})||\text{Sn}*\right\}$ by decrypting ${\text{AID}}_{k}^{*}$. Therefore, it is not possible to impersonate the user.

***Gateway Impersonation Attacks.*** As long as an attacker does not possess the secret key *SK_gs_*, he/she cannot impersonate the server and cannot cheat the sensor node. Hence, it frustrates attackers to generate the valid message *M4* to the sensor node.

***Insider Attacks.*** It is possible in a real-time environment, when the gateway manager or system administrator can use the user password *PW_k_* (*e.g.*, *weak password*) to impersonate the user *U_k_* through any other network gateways [20,21,40]. In this case, our scheme does not give any room for privileged insiders, since, in the registration phase, the user *U_k_* is passing *h*(*r⊕PW_k_*) instead of the plain password. Thus, the insider of the *GW* node cannot get *PW_k_* easily [20,21]. Here, *r* is a sufficiently high entropy number, which is not revealed to the *GW* node. Furthermore, the proposed scheme does not store any verifier table and can resist the insider attacks [41].

***Stolen-Verifier Attacks.*** The stolen-verifier attack scenario is not applicable to our scheme, as we are not using any password/verifier table.

***Offline-Password Guessing Attacks.*** Our scheme is free from any password verifier table, so password guessing attacks are not feasible. In the login phase, passwords are not simply transmitted, instead, they are transmitted with some other secret (*i.e.*, *V_k_* *= h*(*ID_k_|| h*(*r⊕PW_k_*))), which makes it difficult to guess the user’s password.

***Man-in-the-Middle Attacks.*** An attacker may attempt a man-in-the-middle (*MIMT*) attack by modifying the login message *<B_k_*, *AID_k_*, *Tu> into <* $B_{k}^{*}$, ${\text{AID}}_{k}^{*}$, Tu*>. However, this malicious attempt will not work, as the false ${\text{AID}}_{k}^{*}$ will not be verified at the *GW* node and the *GW* node cannot get the original sub-message $\left\{h({\text{ID}}_{k}^{*})||\text{Sn}*\right\}$ by decrypting ${\text{AID}}_{k}^{*}$. Thus, man-in-the-middle attacks are not applicable to the RUASN scheme.

***Secure Password Update.*** In the secure password update phase, our framework first verifies the old *ID_k_*, *PW_k_* and only then requests a new password. Otherwise, it rejects all password update requests. Therefore, our framework updates passwords securely.

***Gateway Secret Key Guessing Attacks.*** In our scheme, the gateway secret keys (*x* and *y*) are very long and possess high entropy. In addition, neither *x* nor *y* are transmitted in plain text over the public channel, instead *x* and *y* are mainly used as a key to encrypt data (*Ex[IDk||l]*, *SKgs = h*(*Sn||y*)). Hence, it is very difficult to guess both the gateway master keys, *x* and *y*.

### 6.2. Efficiency Analysis

In this subsection, we present an efficiency analysis (*i.e.*, *computational cost* and *communication cost*) of our scheme and compare it with existing schemes (Das [18], He *et al.* [21], Wong *et al.* [10] and Vaidya *et al.* [14]) for wireless sensor networks. The evaluation parameters are shown below:

***H***: performing one-way hash function.
***S***: symmetric cryptosystem.
***MAC***: the time for performing a *MAC.*

***Computation Cost:*** *RUASN* adopts low-cost computations like a one-way hash function and symmetric cryptosystem, which is acceptable for WSNs and provides more security features with reasonable computational costs. As we can see the computation cost comparisons of our scheme and other related scheme are summarized in Table 4. It is easy to see that, in the registration phase (*i.e.*, *one-time job*) our scheme requires *4H* and one symmetric cryptosystem, whereas in [21] and [14] *6H* and *4H* are required, respectively. Furthermore, in the login and authentication phase the proposed scheme requires *9H*, *6S* and *2MAC*, whereas, [18,21] and [14] require *9H*, *11H* and *9H*, respectively. This is due to fact that in order to provide more functionality such as mutual authentication, user anonymity, message confidentiality, and secure session key establishment, more computational costs are incurred.

***Communication Cost:*** It is easy to visualize from Figure 3 that *RUASN* requires four message exchanges for the whole communication and confirmation of all entities (*i.e.*, *user*, *gateway* and *sensor*), which is practical for real-time applications.

### 6.3. Functionality Analysis

From Table 5, it is easy to see that the *RUASN* has more security functionality as compared to other existing proposed protocols for WSNs. Our scheme has robust security features such as mutual authentication between all entities (*i.e.*, *user*, *gateway* and *sensor*), user anonymity, confidentiality, secure session key establishment, secure password update phase and secure against insider attacks, and it meets all the requirements of Liao *et al*. [27], which are discussed in Section 3.

As we have seen in the above analysis, it is clear that the *RUASN* is a robust user authentication protocol and provides more security services at less cost.

## 7. Conclusions

In real-time, as the sensor networks themselves offer services to users; it is necessary to control who is accessing the information and if it he/she allowed to do so. Therefore, access control is an imperative requirement for wireless sensor networks to protect the data access from unauthorized parties.

In this regard, we have proposed a robust user authentication framework for wireless sensor networks, RUASN, which is based on a two-factor approach (*i.e.*, *password* and *smart card*) by exploiting the advantages of cryptographic hash functions and cryptosystems. We have shown a security analysis and performance analysis of the RUASN framework and compared it with recent existing schemes. Through analysis, we show that our scheme is more robust against many popular attacks, which are prominent risks for wireless sensor network and that it provides many security services (*i.e.*, *mutual authentication*, *user anonymity*, *confidentiality*, *secure session key and allow users to choose/updates their password*) at reasonable computational costs.

## Figures and Tables

**Table 1.** Notation and symbols used in the paper.

**Notation**	**Descriptions**
*GW* node	WSN gateway node
*U_k_*	*k*^th^ User to be login
*ID_k_*	Login_ID of *U_k_*
*PW_k_*	Password of *U_k_*
*x and y*	Gateway master keys
*r*	Arbitrary random number selected by user
*l*	Random number generated by the *GW* node
*E_key_[m]*	Message *m* is encrypted with symmetric *key*
*D_key_[m]*	Message *m* is decrypted with symmetric *key*
*MAC__key_* (*m*) [31]	Message authentication code over message *m* with secret *key*
*S_n_*	Sensor Node *ID*
*X_g_*	Secret parameter generated by the user *U_k_*
*h*(.)	Cryptographic hash function
⊕	Bitwise XOR operation
||	Concatenation operation

**Table 2.** Additional notations.

**Notation**	**Description**
*U*, *GW* and *Sn*	User, Gateway and Sensor, respectively entities
*X1→X2*	X1 replace by X2
*Phase - I*	Registration phase
*Phase - II*	Login phase
*Phase - III*	Authentication phase

**Table 3.** Local Sets for RUASN.

***1.******Entity U***
*POSS(U) = {PW_k_*,*{ID_k_}*,*r }*
*BEL(U) = {#(PW_k_)*, *#(r)}*
*BL(U) =*
(1.1) Phase – I
▪ *Hash(h(.); XOR(r*, *PW_k_))→Pass* ▪ *Send(GW*, *{ID_k_*, *Pass})* ▪ *Update({ID_k_*, *PW_k_*, *r})* ▪ *Receive (GW*, *{A_k_*, *B_k_*, *V_k_*, *h(.),E_k_, D_k_})*
(1.2) Phase – II
▪ *Hash(h(.);Concat(ID_k_*, *Pass))→*$V_{k}^{*}$ ▪ *Check(*$V_{k}^{*}$, *V_k_)* ▪ *Hash(h(.); A_k_)→H_k_* ▪ *Generate-secret(X_g_)* ▪ *Concat(Tu*, *H_k_)→Tk_Tu_* ▪ *Encrypt({Concat(Hash(h(.);ID_k_*,*Sn*, *X_g_))}_TkTu_)→AID_k_* ▪ *Send(Sn*,*{B_k_*, *AID_k_*, *Tu})→M1* ▪ *Update(ID_k_*, *X_g_*, *Sn*, *Tu)*
(1.3) Phase – III
▪ *Receive(Sn*, *{L*, *Ts})→M4 [Here L=E_SesK_[X_g_||Ts]]* ▪ *Split({L*, *Ts})* ▪ *Check-freshness (Tu* – *Ts)* ≥ Δ*T*, *if yes*, *then aborts.* ▪ *Hash(h(.);Concat(Hash(h(.);ID_k_*,*X_g_*,*Sn*,*Ts*, *Tu)))→SesK* ▪ *Decrypt({L}_SesK_and obtain (*$X_{g}^{*}$, Ts*)
*Check(X_g_*, $X_{g}^{*}$)
▪ *Check(Ts*, Ts*)
***2.******Entity GW***
*POSS(GW) = {x*, *y*, *l*, *and SK_gs_}*
*BEL(GW) = {#(s)*, *#(y)*, *#(l)*,*#(SK_gs_)}*
*BL(GW) =*
(2.1) Phase – I
▪ *Receive(U*, *{ID_k_*, *Pass})* ▪ *Generate-secret number(l)* ▪ *Hash(h(.); XOR(ID_k_*, *l))→A_k_* ▪ *Encrypt({Concat(ID_k_*, *l)}_x_)→B_k_* ▪ *Hash(h(.);Concat(ID_k_*, *Pass))→V_k_* ▪ *Send(U*, *{A_k_*, *B_k_*, *V_k_*, *h(.)})* ▪ *Update({A_k_*, *B_k_*, *V_k_*, *h(.)*, *Pass})* ▪ *Forget({A_k_*,*B_k_*, *V_k_*, *Pass})*
(2.2) Phase – II
*NA*
(2.3) Phase – III
▪ *Receive(Sn*,*{M2*, *Q})[Here M2 = B_k_*, *AID_k_*, *Tu*, *Ts*, *Sn]* ▪ *Split(M2*, *Q)* ▪ *Split(M2)* ▪ *Check-freshness (Tg* – *Ts)* ≥ Δ*T*, *if yes*, *then aborts.* ▪ *MAC({B_k_*, *AID_k_*,*Tu*, *Ts*, *Sn}_SKgs_)→Q′* ▪ *Check(Q*, *Q′)* ▪ *Decrypt({B_k_}_x_) and obtain [*$ID_{k}^{'}$, *l′]* ▪ *Hash(h(.);*$ID_{k}^{'}$*)* ▪ *Hash(h(.);XOR(*$ID_{k}^{'}$, *l′)) →*$A_{k}^{'}$ ▪ *Hash*$(h(.);A_{k}^{'})\to H_{k}^{'}$ ▪ *Concat*$(Tu,\;H_{k}^{'})\to Tk_{Tu}$ ▪ *Decrypt({AID_k_}_TkTu_) and obtain[h(*$ID_{k}^{*}$), Sn*, *X_g_]* ▪ *Check(h(*$ID_{k}^{*}$), *h(*$ID_{k}^{'}$*))* ▪ Check(Sn*, *Sn)* ▪ *Encrypt({Concat(Hash(h(.);ID_k_)*, *X_g_*, *Tg)}_SKgs_)→C* ▪ *Send(Sn*, *{C*, *Tg})→M3* ▪ *Update(C*, *Tg)*
***3.******Entity Sn***
*POSS(Sn) = { SKgs*, *Sn }*
*BEL(Sn) = {#(SKgs)*, *#(Sn)}*
*BL(Sn) =*
(3.1) Phase – I
*NA*
(3.2) Phase – II
▪ *Receive(U*, *{M1}) [Here M1= (B_k_*, *AID_k_*, *Tu)]* ▪ *Split(M1)* ▪ *Check-freshness (Ts – Tu)* ≥ Δ*T*, *if yes*, *then aborts.* ▪ *MAC({Concat(B_k_*,*AID_k_*,*Tu*,*Ts*,*Sn)}_SKgs_)→Q* ▪ *Send(GW*, *{M2*, *Q}) [Here M2 = (B_k_*,*AID_k_*, *Tu*, *Ts*, *Sn)]* ▪ *Update(M2*, *Q)*
(3.3) Phase – III
▪ *Receive(GW*,*{Tg*, *C})* ▪ *Split(Tg*, *C)* ▪ *Check-freshness (Ts –Tg)* ≥ Δ*T*, *if yes*, *then aborts.* ▪ *Decrypt({C}_SKgs_) and obtain [h(ID_k_*, *X_g_*, Tg*)] ▪ Check(Tg*, *Tg)* ▪ *Hash(h(.);Concat(Hash(.);ID_k_*, *X_g_*, *Sn*, *Ts*, *Tu))→Ses_K_* ▪ *Encrypt({Concat(X_g_*, *Ts)}_SesK_)→L* ▪ *Send(U*, *{L*, *Ts})→M4* ▪ *Update(L*, *Ts)*

*NA:* Not Applicable.

**Table 4.** A performance comparison of RUASN with the existing schemes.

**Schemes**	**Registration**		**Login and Authentication**		
	**User**	**Gateway**	**User**	**Gateway**	**Sensor node**
	
Das [18]	*-*	*3H*	*4H*	*4H*	*1H*
Daojing *et al*. [21]	*1H*	*5H*	*5H*	*5H*	*1H*
Wong *et al*. [10]	*-*	*3H*	*-*	*1H*	*3H*
Vaidya *et al*. [14]	*2H*	*2H*	*3H*	*3H*	*3H*
**Proposed RUASN**	***1H***	***3H + 1S***	***4H + 2S***	***4H + 2S + 1MAC***	***1H + 2S + 1MAC***

**Table 5.** Functionality comparison of RUASN with existing schemes.

**Security Features**	**Das [18]**	**He*et al*. [21]**	**Wong*et al*. [10]**	**Vaidya*et al*. [14]**	**Proposed RUASN**

Provides mutual authentication	No	No	No	Yes	**Yes**
Provide user privacy	No	Yes	No	Yes	**Yes**
Confidentiality	No	No	No	No	**Yes**
Secure Session key agreement	No	No	No	No	**Yes**
Secure password update phase	No	Yes	No	Yes	**Yes**
Replay attack	Yes	Yes	Yes	Yes	**Yes**
No password tables stored inside the gateway	Yes	Yes	No	No	**Yes**
No verification table stored inside the gateway	Yes	Yes	No	No	**Yes**
Password is not be transmitted as plaintext	No	Yes	No	Yes	**Yes**
Resist insider-attacks	No	Yes	No	Yes	**Yes**
Password is not exposed to the gateway administrator	No	Yes	No	No	**Yes**
Secure against gateway secret key guessing attack	No	No	No	No	**Yes**
Secure against password guessing attack	No	Yes	No	Yes	**Yes**
